# Dental Fees and Etiquette

**Published:** 1863-03

**Authors:** A. Benedict


### DENTAL FEES AND ETIQUETTE.

Read before the Michigan Dental Association.

BY H. BENEDICT.

IN every department of life in a civilized community, the *true* man lives and acts as well for the benefit of the human race in general as he does for himself.

If he lives in a community that has had greater advantages than himself, he strives by close application and strict integrity to elevate his condition to their standard. On the other hand, if there are those around him not his equals, he strives to elevate them by impressing on them their privilege and duty of becoming such or even his superior. In either case he is benefitting those around him as well as himself, and is placing his foot upon a solid and high round of the ladder he is attempting to climb. We are social beings, and no man ever advanced in knowledge or morals by excluding himself from his fellow man. We are dependent beings, therefore it is necessary that we help each other, and in so doing, if our aim be for the perfect, we elevate ourselves in the same proportion as we hope to elevate those around us. If we were all perfect, we should see, think and act in harmony; but being imperfect or blind through ignorance, we see, think and act differently—therefore the necessity of the motto of the *Dental Cosmos*, "Observe, compare, reflect, record." It is in the observance of that motto that all knowledge is acquired.

Every thing that we do to advance true knowledge, and bring others to see and embrace it, places us one round higher on the ladder towards perfection. The one who is teaching the child his a, b, c, or demonstrating to the youth a problem in mathematics or geometry, or explaining to the student the laws and affinities of chemistry; and all others who are helping the inquiring mind to advance, are not only benefitting the world, but themselves also. But the mind can not labor here without the body, and the body requires food and raiment to sustain it, and to be kept in perfect order, so that the mind may act in its fullest capacity. Therefore it is necessary that some till the ground, others work as mechanics others as artists, and others as teacher and professors. All can not follow one calling, neither can any one branch become independent and say it has no need of the others. All must work in harmony together, and each be conducted by those whose knowledge and particular training fits them for the part they are taking, in order that the greatest good may be accomplished. Each branch is composed of individuals, and any person who does not belong to some one of the industrial branches is a drone, and the world would be as well if not better off without him. He may have money in abundance, but if he hoards it up and does nothing himself, and keeps his money from the legitimate channels of trade, he is just so much dead weight on society and hinders its advance. The beggar who roves from place to place or sits by the wayside idling away his time, when he is able to be doing any thing to improve his condition, does no more to retard the advance of the world than he who gives him, and thereby encourages him in his idleness. There is also a class who make money their *idol*, and have for their motto, "get all you can, no matter how, providing you do not get into prison." They never give a fair equivalent if they can avoid it, are always taking advantage of the unsuspecting. If they are disgracing a profession, they pretend to some secret or skill that no one else has, and palm off their nostrums or pretended skill on the credulous at any price that will pay best. Some of them are satisfied to just live, others are ambitious to hoard up money to again use for as base purposes as it was obtained. Such persons are not only a clog to society, but actually exert all their influence against its advance. Too many such are found in all the different pursuits of life.

Let us now look at the Dental profession, the one we have chosen, and see how far this applies to us. It is now acknowledged by all intelligent persons, that dentistry is one of the most important professions, and requires the highest moral and intellectual persons, as well as those well skilled in its manipulation, to succeed in performing all its different operations in the highest state of perfection to which it is now brought. Neither must we be satified with what we now know, but be constantly on the search for new truths and should embrace every opportunity to advance our knowledge. We must not shut ourselves in our office and say that we know it all, or stay at home when a dental convention meets that we could attend, because we think there is nothing to learn there, or if there is, we shall see it in the Dental Journals, (if we are not working at so low a price that we can not afford to take one,) for if we do we shall find at the end of a year or two, when we emerge from our shell, that we are just so much behind the times. The man who engages in dentistry should do so with a full knowledge of its responsibility, and the patience, toil and perseverance it will require. He must remember that he is not at work on wood, stone or the metals as a mechanic, but that he is operating for human beings, and on living tissue and sensitive dentine; that the health and comfort of his patient may be affected for good or for evil through life by his skillful or unskillful operations. If through his ignorance and lack of skill or carelessness on his part, he destroys a natural tooth that otherwise could be saved, he has done an injury that all the gold of the world or wisdom of the wise men can undo.

But the quack or non-mechanical dentist will say, "Sir, I can put in an artificial tooth so near like the one destroyed, that it can hardly be detected, and it will answer a good purpose to eat on." Grant it, Sir, that you have acquired mechanical skill enough to select a tooth (made by some artist that is able to imitate nature,) and so arrange it on a plate, that the person can wear it. What claim does that give you to the title of D. D. S., or what compensation is it for the injury you have done. What would you think of the surgeon who, when called upon to prescribe for a sore on your foot or hand, should advise you have it cut off, saying it may be cured, but then another sore may come, and it is cheaper to remove it at once. Then I have such nice ones on hand from which one can be selected and fitted on immediately, and it will last you your life-time; it will never be sore and ache or trouble you again, and they are brought to such perfection you can use them almost or quite as well as the natural one."

Or if he should attempt in the first place to cure it, and for want of a thorough knowledge of his remedies or skill in applying them, the disease be aggravated or allowed to take its destructive course, until to save your life or relieve you of a dead and useless member, you are compelled to have it removed.

Which one of you would have a foot, hand or tooth that could be saved and made useful to you, removed for the sake of wearing an artificial one, or even for the price of all the artificial feet, hands or teeth that ever were made. We do not wish to convey the idea that artificial teeth are never necessary, but, on the contrary, they are of great importance, and when it is necessary to resort to their use, they should be constructed by or under the superintendence of a dentist well informed in that part of his profession. Here, of late years, is the door through which most of the empirics enter, and in most cases the loadstone which drags us down. The quack makes it his hobby, and you can generally tell where he is by the display of one or more sets of teeth in his window or at the side of his door. At other times by his handbills and advertisements, telling the public that he has just arrived from some great city in this or the old country, where he had an unlimited practice; but he had sacrificed it for the sake of letting the people of that place have the benefit of his great talents and skill, and at a far cheaper rate than can be had any where else. And now we come to the main part of our subject, that is, Dental fees and etiquette; and you may ask, "What has all you have said to do with it?"

We will answer. In the first place we have shown that the true man is at work for the good of his fellow beings. Second, that the mind can not labor without the body, and that it must have food and raiment. Third, that it is necessary to divide labor; we can not all follow one calling, therefore we are obliged to exchange our labor for another's, or what is equivalent thereto in dollars and cents. Fourth, that he who idles away his time, and the one who encourages him in so doing by giving him, are alike guilty and are in reality defrauding the rest. Fifth, that a fair equivalent should always be given in return for what you receive. Sixth, that the true dentist stands with the profession one of the highest, and should therefore receive his reward accordingly. Then comes the question, what or how much shall that be? Let us for a moment see what are his claims.

The honest dentist has three things in view, no one of which he can lose sight of, without doing some one or more an injury. First, his profession or brother dentists. Second, his patrons; and third, himself. He is fully aware, to keep up with the rapid advance that is made in dentistry, he has to spend considerable of his time in study, experiments and communication with the other members; or in other words, "observe and compare." In that way has dentistry been ennobled to the position it now occupies, not by one or two, but by the help of all who have had the good of their fellow beings and the ennobling of the profession at heart.

And is it not our duty to be one of that number? Let us remember that the community requires it of us, and that it is for their benefit that we thus labor and toil, spend our time and money to prevent or relieve them of that worst of all pains, an aching tooth.. They also require of us to right that which nature has placed wrong, and restore what disease or accident has destroyed. In a word, they intrust to our care those *pearls* which give the most beautiful expression (when rightly arranged and cared for) to the human face, and in this trust greatly depends their beauty, comfort and happiness for life. Shall we accept the *trust* lightly as a mere trade by which we can live, and endeavor to gain business as a dishonest tradesman—by flaming handbills of some new secret, or going from house to house soliciting patronage, or worse than all, by trying to equalize it with dollars and cents, and putting our price lower than the general standard in our vicinity, thus acknowledging ourselves inferior to the rest. Or shall we accept it as becomes honest men, with a full appreciation of its responsibility, and a determination to spare no pains to be worthy of it, and in so doing take the course that all will acknowledge is the right one. We support our Dental Colleges and Journals; belong to and be a worthy acting member of one or more Dental societies, and advance the interest of the whole as far as possible. And now we come to the rock on which we are most likely to fall and be broken to pieces: that is, self. It often gets before our mind in such a way as to entirely blind us in regard to the duty we owe each other and our patrons, and makes us think that we are benefitting ourselves when we are doing one or the other, or we might say, both an injury; but how false the doctrine—for no person really gains any thing by doing that that will wrong his fellow beings. We owe to ourselves and our's, food, raiment, education and protection, and as we have chosen a branch of business in which we are constantly at work for the good of others, we are dependent on what we receive from our patrons for our support. And who will say when in consideration of the responsibility of our *trust*, and the necessity of our whole mind being engaged in it, that it should not be enough to relieve us entirely of all anxiety on that subject. It should be enough to give us time and means to keep up with the improvements, for our patrons demand it, and we are wronging them if we do not. It should certainly be enough to clothe, feed and shelter ourselves and family in as good a manner as the majority around us, and at the same time lay by something for old age, so that we become not burdens upon our neighbors.

It should be enough to enable us to educate our children, and place them in a position to be useful members of society, or we are doing them injustice. And it certainly should be equal to the majority of the best dentists, and always up to the standard of those near our location, or else we are robbing our brother dentists, degrading our profession and retarding its advance, consequently injuring ourselves and our patrons. Therefore the dentist, who endeavors to get business by lowering the price, should be treated by the whole community as dishonest, and one not to be trusted. And until they are so treated, the world will be filled with quacks and the people cheated. Let us all resolve and carry our resolution into effect, that we will do the best we can and not be contented until we are equal to the best; that we will keep up our reputation and business by our honesty and skill, rather than by the dollars we may ask.

That we will neither wrong the profession, our patrons, or ourselves, by putting our price so low that it will not remunerate us well for all the pains we take for the trust reposed in us, but will enable us and those who follow us to be more competent for the task. And here let me add, that the honest and true dentist will always adopt rules to govern him in his deportment in his office and to his patrons, that will be right and just to all. That the one who rides in his carriage will be no better served than the one who comes on foot, and all treated with the same courtesy, whether rich or poor.

Hoping the time will come when all who are engaged in the Dental profession will be in earnest to accomplish all the good they can, and that they shall see, think and act for the advancement of the whole, I leave the subject for your consideration.

DETROIT, January, 1863.

